# Protecting Breastfeeding during the COVID-19 Pandemic: A Scoping Review of Perinatal Care Recommendations in the Context of Maternal and Child Well-Being

**DOI:** 10.3390/ijerph19063347

**Published:** 2022-03-11

**Authors:** Aleksandra Wesołowska, Magdalena Orczyk-Pawiłowicz, Agnieszka Bzikowska-Jura, Małgorzata Gawrońska, Bartłomiej Walczak

**Affiliations:** 1 Laboratory of Human Milk and Lactation Research at Regional Human Milk Bank in Holy Family Hospital, Department of Medical Biology, Medical University of Warsaw, Litewska 14/16, 00-575 Warsaw, Poland; aleksandra.wesolowska@wum.edu.pl (A.W.); agnieszka.bzikowska@wum.edu.pl (A.B.-J.); 2 Human Milk Bank Foundation, Podkowy Str. 128 J, 04-937 Warsaw, Poland; me.gawronska@uw.edu.pl; 3 Department of Biochemistry and Immunochemistry, Division of Chemistry and Immunochemistry, Wroclaw Medical University, M. Skłodowskiej-Curie 48/50, 50-369 Wrocław, Poland; magdalena.orczyk-pawilowicz@umw.edu.pl; 4 Faculty of Sociology, University of Warsaw, Karowa 18, 00-927 Warsaw, Poland; 5 Institute of Applied Social Sciences, University of Warsaw, Nowy Świat 69, 00-927 Warsaw, Poland

**Keywords:** COVID-19, SARS-CoV-2 infection, breastfeeding, human milk, well-being, perinatal care, recommendations, guidelines

## Abstract

The objective of this scoping review is to determine to what extent the recommendations on perinatal care protect breastfeeding during the COVID-19 pandemic. The review follows the PRISMA ScR Extension guidelines. The research was conducted in Scopus, Medline via Pubmed, and Web of Science databases from 1 March 2020 to 31 May 2021, using 392 combinations of keywords. We searched for reviews and original papers published in English providing recommendations on delivery mode, companion during labor, the possibility of skin-to-skin contact (SSC), breastfeeding, and visitors policy. After screening, 86 out of 8416 publications qualified for data extraction. The majority of them indicated that COVID-19 infection is not a sufficient reason for a cesarean section; however, on a national level, cesarean births in severely ill patients were overrepresented. A significant number of recommendations deprived mothers of the necessary support during their labor and stay in the maternity ward. A shared decision-making model was hardly visible. Only the earliest COVID-19 recommendations suspended direct breastfeeding; in later publications, decisions were related to the mother’s health, but other options of natural feeding were rarely discussed.

## 1. Introduction

Pregnancy and childbirth are recognized as one of the most significant life experiences for women and entire families [1,2,3,4]. Therefore, treatment protocols for pregnant women and clinical recommendations for perinatal care have been family-centered and respect maternal and child well-being. Special efforts have been made for many years to improve the quality of maternal and newborn care in health facilities by promoting and protecting breastfeeding. One of the most recognized and globally disseminated good practices in this regard is the Baby-Friendly Hospital Initiative that was launched by the World Health Organization (WHO) and UNICEF in 1991, following the Innocenti Declaration of 1990 [5]. The main goal of this effort is to improve the role of maternity services worldwide, enable mother–baby bonding, and promote breastfeeding for the baby’s best health and well-being, regardless of economic status or health condition at the time of delivery.

Well-being is defined by the WHO as the mental state in which an individual realizes his or her own abilities, can cope with the normal stresses of life, can work productively and fruitfully, and is able to contribute to his or her community [6]. By applying this definition to pregnancy and childbirth, well-being can be considered access to resources for physical and mental health that enable carrying out tasks, such as childbirth, satisfactorily [7,8]. Critical factors for physical and mental health during delivery include a choice of mode of delivery, openness for companions, close mother and child contact (skin-to-skin contact—SSC, kangaroo care, and rooming-in), ability to put the baby onto the breast, and support for proper latching on [9,10,11]. Upholding this highest standard of mother and baby care was challenging during the outbreak of the COVID-19 pandemic [12,13]. The worldwide spread of a new unknown viral disease caused by SARS-CoV-2 with an initially unknown mode of transmission necessitated a change in the well-established practices of childbirth and perinatal care [14,15,16]. Especially in the first period after the pandemic outbreak, there were thousands of new position papers, interim guidelines, opinions, and recommendations issued by individual experts and bodies, scientific societies, and global public health organizations [17,18]. According to the WHO definition, a guideline is a document including a set of clinical evidence-based recommendations that allows the best possible decision for a patient’s treatment or care. The term “recommendations” may be used more narrowly to refer to specific clinical actions in each field that are based on evidence of varying levels of proof [19,20]. Although the two terms are sometimes used interchangeably, in our text, the term “recommendations” refers to the various proposed maternal and child management approaches that could be found in the scientific literature during the COVID-19 pandemic, without detailed analysis of quality and reliability. We noticed that among the 60,000 publications on maternal and child health and nutrition downloaded in the Johns Hopkins repository between the end of February 2020 and May 2021, only 15 are systematic reviews, and few reviewed guidelines for pregnant women and concerned breastfeeding [21]. Therefore, our objective was to analyze the broad spectrum of recommendations on perinatal care during the pandemic that appeared to be critical to maternal and child well-being and support breastfeeding. Considering the limited state of knowledge and the particular importance of the subject, we decided to use a scoping review framework to form a comprehensive picture of trending perinatal care during the COVID-19 pandemic in regard to breastfeeding protection and mother and child well-being.

## 2. Materials and Methods

The review followed the scoping review extension of Preferred Reporting Items for Systematic Review and Meta-Analysis (PRISMA) guidelines [22]. The entire process was conducted following an unpublished a priori protocol with the Covidence systematic review software (Veritas Health Innovation, Melbourne, Australia, available at www.covidence.org (accessed on 31 January 2022)).

### 2.1. Data Source, Search Strategy, and Inclusion/Exclusion Criteria

We examined Scopus, Medline via Pubmed, and Web of Science databases for the combinations of three sets of keywords. The first set consisted of the words: “delivery”, “labor”, “labour”, “giving birth”, “breastmilk”, “human milk”, “donor milk”, “own mothers milk”, “breastfeeding”, “mother infant contact”, “skin to skin”, “prenatal care”, “postnatal care”, “perinatal care”. The second set included: “recommendation”, “counselling”, “policy”, “guidelines”, “guidance”, “statement”, “best practices”. The third set included the following terms: “COVID-19”, “COVID19”, “coronavirus”, and “SARS CoV-2”. For the research, we used all possible combinations of words from three sets, for example: “delivery” AND “recommendation” AND “COVID-19”, then “delivery” AND “recommendation” AND “COVID19”, “delivery” AND “recommendation” AND “coronavirus”, etc. This strategy resulted in 392 combinations of keywords in total (for a detailed search strategy, see Supplementary Table S1). The time frame was from 1 March 2020 to 31 May 2021. However, some papers were available online before the date of publication and thus met established criteria, even though the official date of publication was later. During the data extraction, the articles were coded using the earliest date of online publication. Inclusion and exclusion criteria are listed in Table 1.

### 2.2. Data Extraction

Relevant information from selected studies was extracted with the predefined data extraction tool. Two independent researchers extracted each paper (MOP, MG, ABJ, or BW); the team discussed disagreements as necessary. Extracted information was of qualitative characteristics and included recommendations on the mode of the delivery, a companion presence, family births, visitor policies, skin to skin contact, rooming-in, breastfeeding, and use of human milk, as well as study characteristics (authorship, country of origin, publication type, the exact date of publication).

### 2.3. Data Analysis

Extracted data were analyzed quantitatively and qualitatively. We provided the frequency of indications and changes in time, coded recommendations into thematic categories, and compared frequencies and the potential influence of recommendations on breastfeeding practice.

## 3. Results

### 3.1. Study Selection

**Figure 1:**
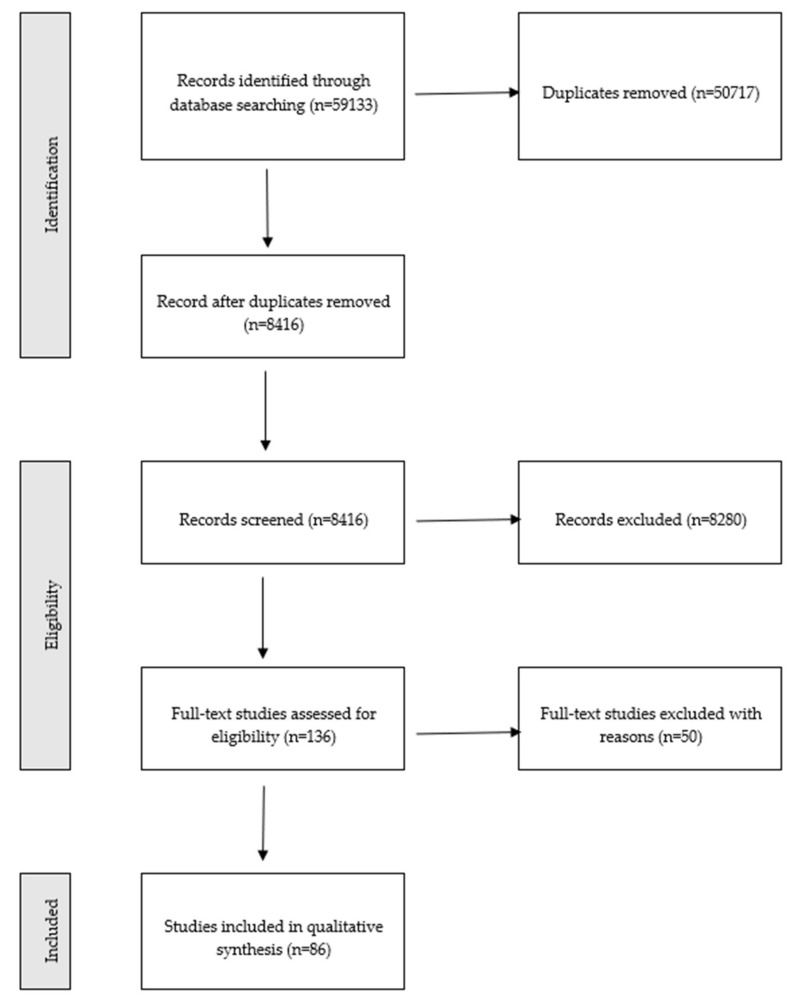
PRISMA flow chart.

The initial search returned 59,133 studies: 10,435 from Scopus, 45,622 from PubMed, and 3076 from Web of Science. However, the majority of identified papers (50,717) were duplicates (Figure 1). Therefore, 8416 proceeded to title and abstract screening. Two independent reviewers (MOP, MG, ABJ, or BW) screened each of the eligible studies. Another pair of reviewers (including AW) solved conflicts by reaching a consensus. For the full-text review, 136 publications were selected. Two independent reviewers (MOP, MG, ABJ, or BW) qualified each study in the full-text review stage. Another pair of reviewers (each including AW) solved conflicts, if necessary. A total of 33 studies were excluded as irrelevant to the topic, 8 because they were in a language other than English, 5 were editorials or letters, 2 were duplicates, the full texts were inaccessible for 2 more. Finally, 86 studies qualified for data extraction (Supplementary Table S2).

### 3.2. Study Characteristics

Half of the studies (43 out of 86) that qualified for extraction were based on a query of original research, typically mixed with references to recommendations issued by the WHO, CDC, and other well-recognized institutions. A total of 23 papers focused on recommendations exclusively without referencing original studies. We also included two systematic reviews which formulated their own recommendations and eight other types of data review. The last group—original research—was relatively small (six papers), as many of the original studies available focused on data description without any recommendations. See Supplementary Table S2 for the complete list of included studies.

More than half of the studies (45 out of 86) did not limit recommendations to any specific country and will be referred to as “non-country specific” in the further analysis. For the country-specific recommendations, we identified seven papers from Italy, six from the USA, five from Brazil, and three from India and Spain. Other countries were represented in one or two publications (Table 2).

### 3.3. Mode of Delivery

Recommendations concerning the mode of delivery were included in 45 out of 86 (52%) of the analyzed publications. Almost 50% (22 out of 45) of available recommendations were non-country specific. For the other half, recommendations were on a national level. Italy had the greatest frequency (four publications), followed by Brazil (three publications), the USA (three publications), and two each in India, Poland, and the UK. The others referred to individual countries, particularly Australia, China, Nigeria, Russia, Saudi Arabia, Spain, and Turkey (see Supplementary Table S3 for details).

**Figure 2:**
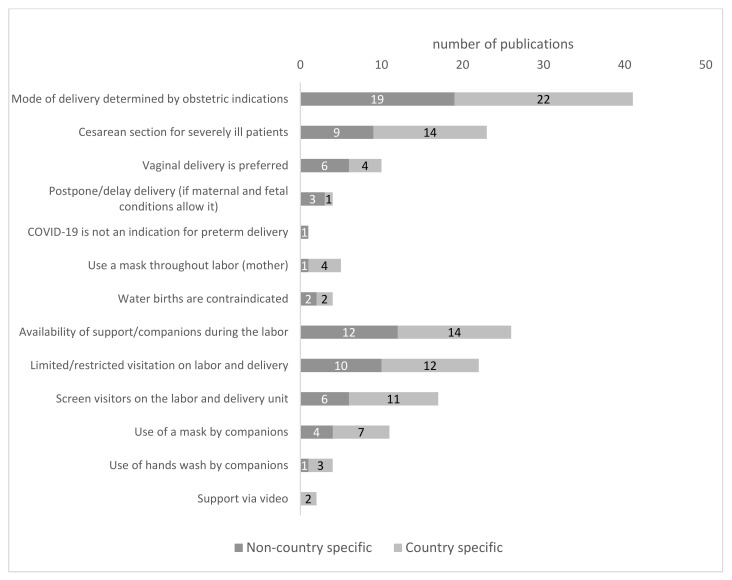
Recommendations concerning the mode of delivery and companions on the labor for mothers with confirmed and/or suspected COVID-19.

Most of the available recommendations, namely 18 out of 22 on a non-country specific and 22 out of 23 on a national level, recommended that the mode of delivery should be determined by obstetric indications only, regardless of maternal COVID-19 status. However, a preference for vaginal delivery or/and no contraindication to vaginal birth was reported in both the non-country specific recommendations [23,24,25,26,27,28] and on national levels [29,30,31,32]. Additionally, two publications suggested that the second stage of labor should be cut short [33,34], including instrumental delivery to be shortened [34] (Figure 2). Of the recommendations with a non-country specific scope, 40% of publications (9 out of 22) recommended cesarean section for patients severely ill with COVID-19 [24,25,26,35,36,37,38,39,40]. In contrast, on the national level, 65% of the available publications (15 out of 23) provided details in terms of the recommendation of cesarean section for severely ill pregnant women [18,30,31,32,33,34,41,42,43,44,45,46,47,48].

Available recommendations regarding the week of delivery in women with confirmed or suspected COVID-19 infection are limited. Only five publications recommended considering delaying the delivery date, but only if maternal and fetal conditions permitted [25,27,45,47,49]. On the other hand, only one publication indicated that COVID-19 is not an indication for preterm delivery [50].

Detailed recommendations concerning the possibility of water birth were provided in four papers; the recommendations were unanimous that water births are contraindicated in the case of mothers with COVID-19 [26,27,51,52].

Of the published recommendations, only one publication issued by a global body [28] and four national ones [32,51,53,54] recommended that a laboring woman’s mouth and nose should be covered with a surgical mask throughout labor.

### 3.4. Rules of Family Births

The availability of companions or relatives during labor was addressed by 26 out of 86 publications included in the analysis. Almost half of the recommendations on support during labor were formulated on a non-country specific level (12 out of 26 publications), whereas on a national level, the frequency of recommendations was the highest for Brazil (3 publications), then Italy, Poland, UK, and the USA (2 publications for each country) (Supplementary Table S3).

Among analyzed recommendations, only one paper [43] (Nigeria) expressly forbids the partner to be present for support during childbirth, and one paper [55] (non-country specific) restricted the choice of an attendant to the child’s father (Figure 2).

The remaining recommendations, 6 made at the non-country specific level [25,26,36,38,39,56] and 11 at the national level [29,30,42,44,45,47,48,52,53,57,58], allowed for family births with 1 person arranged by the mother. Making the possibility of family birth dependent on local conditions and leaving the final decision to the authorities of each hospital were included in four papers, one on the non-country specific level [49] and three on the national level [23,24,53]. Moreover, one paper recommended no visitors or one support person [59].

When support and companions during delivery were allowed, the authors recommended additional specific requirements for such persons, independent of the general pandemic restriction in hospitals, such as wearing masks (as highlighted as obligatory for companion persons in three papers on a non-country specific level [25,26,39] and six on a national level) [30,44,47,51,53,54] as well as washing hands (stressed in one of the recommendations issued non-country specific [38] and one [29] country-specific). Moreover, an obligatory screening test for COVID-19 of a support person during labor and delivery was recommended in 13 publications [29,30,38,39,42,45,48,51,53,55,56,58,59]. Less restrictive conditions, namely that the accompanying person must be asymptomatic without the need for a test, were recommended in three publications on the non-country specific level [25,26,36] and three papers on the national level [44,52,54] (Figure 2).

### 3.5. Visitors Policies

Recommendations on openness to newborn visitors were indicated in 43% (37 out of 86) of papers included in our study. About half (19 out of 37) of the recommendations on visiting a newborn were formulated on a non-country-specific level. Other publications directly referred to particular countries, most often the USA and Brazil (three publications each). The rest of the countries were mentioned by single papers (see Supplementary Table S4). A total of 29 articles were published in 2020 (no later than in October 2020), another 8 were published in the first half of 2021.

**Figure 3:**
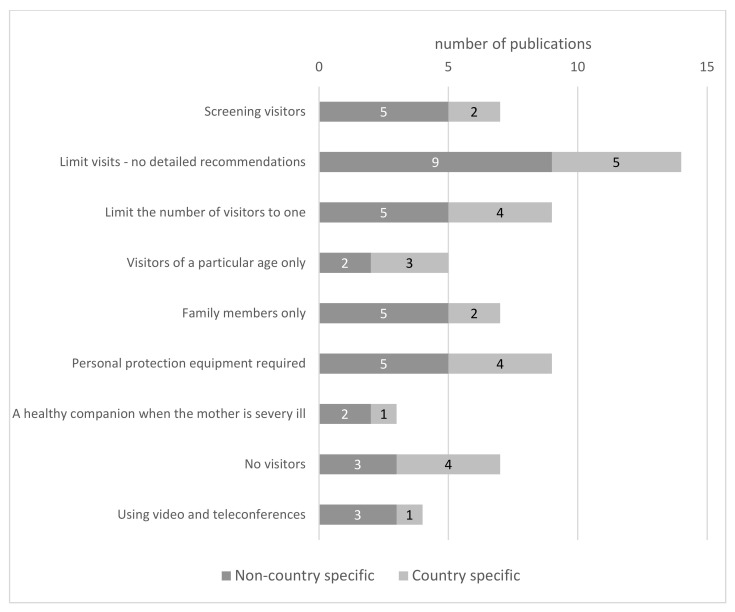
Recommendations concerning visitor policies on maternity wards for mothers with confirmed and/or suspected COVID-19.

A total of 20 publications recommended specific policies for visitors, while only 14 gave general recommendations (“limit visits”) (Figure 3). The two most common were limiting the number of visitors to one [25,48,54,56,58,60,61,62,63] and using personal protective equipment (PPE) [25,27,28,47,63,64,65,66,67]. It is noteworthy that all the recommendations that did not allow more than one visitor were published within the first three months of COVID-19, except Boeling’s second article from October 2020 [48]. Seven studies [39,41,53,55,56,68,69] recommended banning all visiting. These recommendations were dispersed over the period under review. They were issued both on non-country specific [39] and on a national [53] level, over one year after the spreading SARS-CoV-2 infection was recognized as a pandemic. Five of the publications [39,55,56,68,69] suggested prohibiting visits only when a child or the mother was suspected of SARS-CoV-2 infection or was already diagnosed with COVID-19. Moreover, three other papers recommended visits only when the mother’s illness did not allow her to take care of the newborn [49,50,66]. When visits were allowed, the authors recommended additional specific requirements. In seven articles, we found further conditions to be met by visitors, mainly related to the degree of relationship to the mother [25,28,49,60,61,65,67], the majority of which were published no later than October 2020. Four [49,60,61,67] limited visits to family members, not indicating the exact relationship with the mother, two recommended the father only [28,65], and one restricted visitors to people living in the same household [25]. Five publications [25,28,30,58,68] included requirements on visitors’ age. A total of 3 papers excluded children [28,30,58], 1 excluded [68] adults over 60 years old, and 1 [25] limited visits to adults aged between 18 and 59 years. Universally testing visitors for SASR-COV-2 infection was recommended in seven publications [25,28,48,56,61,63,65]. Four papers proposed using video and teleconferences as an alternative to direct visits to labor and delivery units [64,65,70,71].

### 3.6. Skin-to-Skin Contact and Kangaroo Care Intervention

Among 86 analyzed publications, 56 (65%) addressed recommendations regarding skin-to-skin contact (SSC), making it the third most popular area identified. Of these, 32 recommendations were formulated on a non-country specific level and 24 on the national level, with the greatest frequency in Brazil (5 studies) and the USA (4 studies), followed by Spain (3 studies). Other countries are listed in Supplementary Table S5. The studies were first published between April 2020 and April 2021. More than half of the studies (30 out of 56) were published in the second and third quarters of 2020, 8 studies in the fourth quarter of 2020, 10 studies in the first quarter of 2021, and 8 in the second quarter of 2021. However, in the initial phase of the pandemic, the practice of SSC was slightly less often addressed than in it was later on. In the second and third quarters of 2020, 64% (16 studies) and 67% (14) of all studies collected and published referred to SSC, while in the fourth quarter of 2020, the number increased to 62% (8 studies), and in the first 2 quarters of 2021, it was 83% (10) and 74% (8) (Supplementary Table S5).

**Figure 4:**
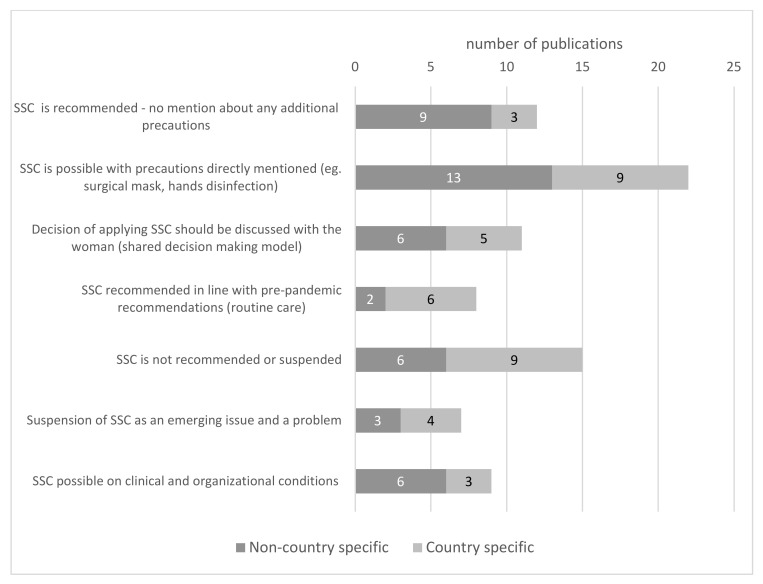
Recommendations concerning skin-to-skin (SSC) contact for mothers with confirmed and/or suspected COVID-19.

Most of the SSC recommendations applied to mothers with COVID-19 and those suspected of SARS-CoV-2 infection (patient under investigation, PUI). Only 4 of 56 papers [18,55,64,72] addressed SSC specifically for mothers with confirmed infection (Figure 4). In general, we observed that 70% (39 out of the 56) of analysed publications concluded that SSC should be allowed or recommended [17,18,27,32,36,42,44,47,49,51,54,55,57,59,60,65,66,69,70,71,73,74,75,76,77,78,79,80,81,82,83,84,85,86,87,88]. In 19% (7 papers) it was directly indicated that, regarding SSC, pre-pandemic recommendations should not be changed and routine care should be provided [18,42,47,69,82,86,87].

Recommendations against SSC were found in 27% of the papers addressing this issue (15 papers) [25,26,28,29,30,37,48,53,61,62,64,68,89,90], including 1 paper in which different kinds of Brazilian recommendations, mostly prohibiting SSC, were recalled and discussed due to being controversial and potentially negatively impacting breastfeeding success, but the authors did not formulate their own recommendations [91]. Half of the recommendations (7 studies) among those against SSC were published in the second and third quarters of 2020 [25,26,37,61,62,64,68].

Most common recommendations allowed SSC with precautions like wearing a surgical mask by the mother as well as hands and surfaces disinfection. This was recommended in 39% (22 out of 56) papers [17,18,27,32,36,49,51,54,57,59,60,66,69,71,73,74,75,76,79,80,85,86]. However, in one paper [86] washing the mothers’ chest before contact with the child was specifically excluded from recommended precautions. A total of 21% papers (12) recommended SSC without directly mentioning any additional precautions that should be taken [42,47,55,65,70,77,78,81,82,83,87,88].

Nine papers suggested that the possibility of practicing SSC should depend on clinical and organizational conditions, meaning that the decision should be individualized in each case [29,36,44,54,60,71,79,92], and especially reconsidered when the mother’s condition is critical [74]. About one-fifth of the papers highlighted the importance of a shared decision-making model and informed choice made by parents and indicated that decisions on applying SSC should be discussed with the mother [29,35,47,54,55,63,71,72,81,84,92] (Figure 4).

### 3.7. Rooming-In

We identified 58 papers among 86 that included recommendations on rooming-in. Half of them were non-country specific, and half were on a national level. The highest number of publications on a national level came from Italy (five), the USA (four), then from Brazil, India, and Spain (three each). Two papers provided recommendations for Poland and Australia, and one each for China, Nigeria, Russia, Saudi Arabia, Turkey, and the UK (Supplementary Table S6).

The papers were published between March 2020 and April 2021. Apparently, the counseling on rooming-in was more popular at the beginning of the pandemic; more than one quarter (16 papers) were dated within 3 months of the WHO declaring the global threat of SARS-CoV-2. In total, three-quarters of recognized papers appeared in 2020.

**Figure 5:**
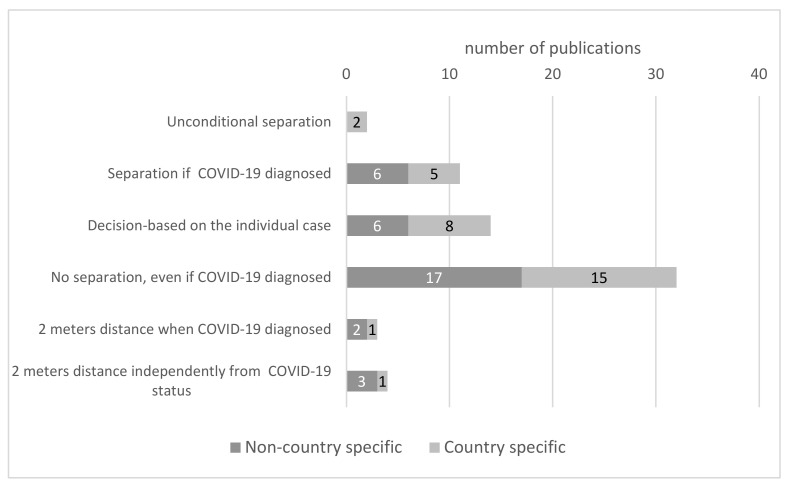
Recommendations concerning rooming-in management for mothers with confirmed and/or suspected COVID-19.

The most frequent recommendation was not to separate a mother from a child unless the mother was too ill to care for their newborn (Figure 5). Rooming-in with neonates positively diagnosed with COVID-19 was acceptable. This recommendation was found in 17 papers on a non-country specific level [25,36,49,50,55,59,60,62,65,70,76,78,79,81,82,83,88] and 15 on a national level [18,32,42,44,47,53,54,61,66,67,68,75,86,93,94]. The publications are dispersed throughout the study period. The earliest comes from April 2020, the next 9 from May and June 2020, so even though guidelines from the WHO and CDC were contradictory at that time, the suggestion to maintain rooming-in was clearly visible.

At the opposite extreme, only two papers recommended unconditional separation. None of them was published on a non-country-specific level. The publications came from the first three months of the pandemics and referred to China [31] and Russia [45]. A total of 12 publications (6 of them on a non-country specific and 5 on a local level), published between March 2020 and February 2021, recommended separation when the mother or the newborn had SASR-CoV-2 infection confirmed [27,28,30,35,40,58,61,64,77,95,96]. However, it should be noted that three papers recommend separation only when the mother is too sick to take care of the neonate [35,58,96]. Chawla et al. [61] suggested not to rooming-in when both mother and child are positively diagnosed with COVID-19.

There were six non-country specific [24,26,71,74,89,92] and eight national recommendations [34,41,46,66,84,85,87,97] that recommended decisions based on individual cases. Some of them stress the need to weigh the benefits and risks of rooming-in while COVID-19 infection occurs [46,97] while Pountoukidou et al. [74] and Mostafa et al. [66] give a decisive voice to the mother.

A minority of recommendations concerned details of rooming-in management to minimize the risk of SARS-CoV-2 infection. Two papers on a non-country specific level [35,71] recommended keeping a distance between the mother’s bed and the cradle plus the use of masks [71] when one of the dyads is positively diagnosed with COVID-19. Similar recommendations may be found in one national-level paper [30]. Two meters distance independent of a diagnosis was proposed in three publications on a non-country specific [28,62,92] and one on a local [29] level.

### 3.8. Breastfeeding and Use of Human Milk

Most of the reviewed papers (73 out of 86, 85%) included recommendations concerning breastfeeding, 23 of 73 addressing this issue (32%) allowed the feeding of babies by expressed mother’s milk (EMM) when breastfeeding is not possible. Not many papers (22, 30%) provided information about donor human milk (DHM) as an alternative in the absence of EMM because of COVID-19. In 39 cases, recommendations were non-country specific, and 34 publications were on the national level. Of the latter, most were in the following countries: Italy (six), Brazil (six), the USA (five), Spain (three), and India (three). Detailed information is presented in Table 3.

Irrespective of maternal COVID-19 status, direct breastfeeding was recommended in most of the revised publications concerning this issue (65 out of 73, 89%), and in 37, we found additional information regarding taking particular precautions by the mother, like respiratory hygiene during breastfeeding (using a medical mask) and washing hands thoroughly with soap or disinfectant before and after each feeding. One of the Brazilian papers [68], although it recommended breastfeeding, also provided some additional information concerning the prohibition of breastfeeding in the first hour after birth. In Nigeria [87], direct breastfeeding was recommended only in asymptomatic mothers and those with mild symptoms (Table 3).

In seven papers [24,31,34,40,64,77,95], the authors indicated that in COVID-19 confirmed mothers, direct breastfeeding is not recommended, and all of them, except one [34], was published in the first half of 2020. Additionally, Chen et al. [31] concluded that direct breastfeeding should be suspended for at least 14 days.

We found that 10 publications [23,46,54,62,63,70,75,79,89,98] supported using expressed own mother’s milk to feed newborns from asymptomatic and suspected mothers or those mothers who personally did not decide to breastfeed. In another six papers [25,34,61,64,84,95], feeding EMM was recommended by a healthy caregiver. In India [67], Italy [42,96,97], and Nigeria [43], only mothers who were too ill to breastfeed or when temporary separation was needed, were advised to feed their child with expressed breastmilk. Similar recommendations were found in two papers on the non-country-specific level [26,38].

Information about the use of donor milk when own mother’s milk was not available was found in 15 papers [17,24,29,40,42,62,64,67,70,71,75,79,88,89,96] and another 6 provided only information about the necessity of the DHM screening and/or tighten up recruitment criteria for potential donors in the time of pandemic [61,63,72,77,80,99].

Recommendations for feeding with infant formula when the mother is SARS-CoV-2 infected were mentioned in three more papers [66,70,87], whereas Haiek et al. [70] underlined that it is the last of the recommended options (after EMM and DHM).

## 4. Discussion

Over the past decade, hospitals across the world have increasingly moved forward to offer a safe environment for mother-baby dyad, not only to survive childbirth but also to give mother–child a sense of confidence and support throughout the entire process—labor and delivery, postpartum, breastfeeding, and beyond [105]. Avoiding separation of the mother–baby dyad, limiting unnecessary treatments, ensuring intimacy, and being with close family members during labor are essential for the well-being of both. In addition, not being forced regarding the mode of delivery and making informed decisions about baby feeding are principles of maternity care that have been widely accepted [106].

The COVID-19 pandemic crisis put the whole healthcare system, including perinatal care to a severe test. Changes in perinatal care have been driven by the goal of minimizing the risk of SARS-CoV-2 mother-to-child transmission and the spread of the disease in hospitals [12,13,14,15,98,107,108]. This study analyzed the adaptation of globally issued and country-specific perinatal recommendations during the COVID-19 pandemic in the context of ensuring maternal and child well-being and breastfeeding protection.

Considering the mode of the delivery during the COVID-19 pandemic, recommendations were found to be generally the same on both non-country specific and national levels; however, for low-income countries, concerns have been raised about the applicability of the recommendation. Confirmed or suspected COVID-19 status alone in most recommendations was not an indication for cesarean section (CS), except in cases in which there were absolute obstetric indications and health reasons related to SARS-CoV-2 infection. Both on the non-country specific and national levels, preferential vaginal delivery (VD) or no contraindications to VD were recommended (Figure 2). As was previously reported [109,110], planned and emergency cesarean section substantially adversely impacted the initiation and continuation of breastfeeding. So, it is particularly important to avoid performing a cesarean section when it is not clinically indicated in terms of breastfeeding and strengthening the immature immune system of the newborn, thanks to bioactive factors present in breast milk. Upholding current recommendations in this field are based on reliable knowledge and the results of scientific research indicating a minor risk of virus transmission during vaginal childbirth if all the rules of the sanitary regime are followed. Moreover, research has shown that the risk of a SARS-CoV-2 positive newborn was even slightly increased after a cesarean section birth (2.7% VD vs. 5.5% CS) [111]. Recommendations regarding the mode of delivery were not always in compliance with the recommendations on the availability of companions of relatives during labor. In the analyzed cohort of papers, only 12 out of 22 on the non-country specific level and 14 out of 23 on the national level provided detailed recommendations in that aspect. Few included specific recommendations on the course of family childbirth except those that made it dependent on the decision of the hospital authorities [53]. Likewise, it appears that visitors’ policy might be considered a subject for the hospitals’ broader visitors management policy during the pandemic, not limited to neonatal and obstetric wards. The recommendations on visiting mothers and newborns were relatively rarely found and typically published within the first six months after COVID-19 was recognized as a global pandemic (Supplementary Table S4). Banning visitors in the name of limiting the spread of SARS-CoV-2 infection by avoiding social contact is understandable during the COVID-19 pandemic. However, the role of those accompanying the mother during labor and the postpartum period is significantly greater than simply providing companionship. Many studies have proven that having a labor companion and postpartum support improves outcomes for women. Finally, the babies of these women are less likely to have a low five-minute Apgar score [105]. Based on this reason, companions of choice during labor and postpartum have been recommended by the WHO since 2012 [112]. Moreover, the possibility of having a companion of choice during labor and childbirth can also be considered from the perspective of basic human rights, especially the right to the highest attainable standard of health, but also an important factor influencing ensuring women’s dignity in maternity care [112,113].

Some of the recommendations also did not respect the mothers’ freedom of choice in terms of the companion by limiting visitors to the husband. To make matters worse, maternal deprivation of psychological and physical support from relatives was held up in recommendations issued in 2021, indicating that this very restricted policy (no visitors or one support person) was not due to over-reacting in the early stage of the pandemic [59].

At the beginning of the COVID-19 pandemic, there were very few recommendations at both the global and national [25,27,45,47,49] levels to delay delivery (if maternal and fetal conditions allow it, for both confirmed and suspected infection of pregnant women. As the pandemic continued and more evidence-based medical data became available, delaying childbirth simply because the mother was infected with COVID-19 no longer was recommended. A similar position concerned the acceleration of labor due to the infection of the pregnant woman. A recommendation that COVID-19 is not an indication for preterm delivery was pointed out only in one paper, published by Api et al. [50]. However, early delivery was indicated as warranted if the pregnant woman was in critical condition, as noted in most global and national recommendations. It seems that the timing of delivery of patients with a positive SARS-CoV-2 test result can be rescheduled considering the severity of COVID-19 infection and obstetric indications such as gestational age and fetal well-being, maternal cardiac disease, diabetes, preeclampsia, and existing comorbidities. Clinical data including over 42,000 pregnant women indicated that the ratio of cesarean to VD was higher (53.2% CS vs. 41.5% VD) in those populations and that pregnant women who were exposed to a heavier course of COVID-19 were more likely to have a risk of preterm delivery [114].

Unfortunately, the analyzed available recommendations did not include details on the possibility of using the bathtub, at least in the initial stage of labor, the choice of which by women giving birth was significant before the pandemic. Of particular importance are the rare recommendations regarding the support of mothers during childbirth thanks to the use of image transfer techniques, namely by video [45,58]. It has recently been proved that watching virtual reality videos reduces stress and tension during childbirth, shortens its length, and strengthens satisfaction [115]. Therefore, there should be an individualized, dedicated approach that provides support to pregnant women during this unique and difficult period and reduces stress and anxiety, which certainly translates into psychological support for mothers, which is important during the COVID-19 pandemic.

The ability for newborns to stay with their mothers unrestricted by rooming-in is the most significant advance for modern mothers and childcare [116]. The benefits of rooming-in are proven for both healthy newborns and vulnerable infants and include facilitating attachment between mother and infant, emotional stability, protection from infection, and increasing breastfeeding rates by making it easier to feed on demand [117,118,119].

However, treating mother and child as an inseparable dyad implies many different doubts for neonatologists, midwives, and obstetrics, especially when we are not dealing with physiology on delivery and postpartum, such as during the COVID-19 pandemic. As a result, it is not surprising that rooming-in is at the heart of the recommendations made during the COVID-19 pandemic. However, early inconsistency in recommendations made by global authorities, such as the WHO and CDC, was the subject of discussion and controversy [59].

The current state of knowledge regarding SARS-CoV-2 infection in the first few days of life indicates that sharing the room of a COVID-19-affected mother and infant and taking precautions minimizes the risk of infection and provides more health and emotional benefits than potential harm [76,114].

Interestingly, despite the confirmed route of SARS-CoV-2 infection through close contact, which is an immanent feature of the rooming-in system, most of the recommendations, including those issued at every stage of the pandemic, recommended rooming-in, even if the mother was diagnosed with COVID-19 (Figure 5). The only reason for limiting mother–child contact, according to the recommendations, was the mother’s inability to care for the child due to illness. The unconditional separation that was proposed in the Chinese and Russian recommendations was quickly judged as possibly harmful to mothers and newborns, especially for initiating and maintaining breastfeeding [76,120].

The rooming-in circumstance is also very important for successful kangaroo care intervention practice for preterms. The ten steps for successful breastfeeding from Baby-Friendly Hospital Initiative (BFHI), WHO/UNICEF, recommends early breastfeeding within 30 min after birth with complete rooming-in care for the first 24 h [121].

It was estimated that keeping mothers and babies together could save more than 125,000 children in 127 low and middle-income countries every year by implementing kangaroo care to reduce preterm deaths by as much as 40%, hypothermia by more than 70%, and severe infections by 65%. At the same time, the risk of death for newborns infected with SARS-CoV-2 could result in the loss of 1950 babies’ lives yearly in the most severe scenario. Hence, the benefit of kangaroo care is 65-fold higher than the mortality risk of COVID-19 [122].

Unfortunately, only eight of the analyzed recommendations referred to the practice of kangaroo care, which shows that the importance of this intervention is still underestimated. The authors of three of them highlighted those recommendations regarding kangaroo care in the case of COVID-19 infected mothers, which were conflicting and diverse [57,59,81]. Five recommendations [17,42,47,67,81] were recommended in favor of kangaroo care, but one [67] recommended engaging another healthy family member in kangaroo care if the mother is confirmed SARS-CoV-2 infected. Only one paper [81] recommended a shared decision-making model and leaving the decision to parents’ informed choice.

Despite the many advantages of complete rooming-in care, including intimacy and frequent kangarooing, a few authors of the recommendations for the COVID-19 pandemic suggested some limitations for maternal illness. These include limited physical contact and the use of screens. It is questionable whether two meters allows the mother to correctly read the needs of the child, building a safe bond and fostering a sense of maternal empowerment and competencies. In fact, full rooming-in requires the infant to either share the mother’s bed, be in a sidecar attached to her bed, or be in a crib right next to her bed, but room-sharing may help mitigate stress related to SARS-CoV-2 infection and calm the mother down [86]. However, the involvement of the mother in the decision making would be a solution to respect the mother’s will, which was relatively rarely noticed in the analyzed part of the studies; it appeared in 14 of 86 papers in the context of a decision about baby separation (see details in Supplementary Table S6).

The same applies to the issue of SSC, which is known as the most effective intervention to ensure the best possible start in life. The opportunity to decide about this important perinatal experience based on current knowledge of the benefits and risks of SSC in the setting of COVID-19 infection was left to the mother in 20% of the analyzed recommendations (Figure 4). It is known that babies held in SSC were more likely to have been breastfed successfully during their first latch on, had higher blood glucose levels, and had a more stable body temperature. In low-income countries, SSC is recognized as a life-saving intervention and as a non-pharmacological intervention for pain control in infants, increasing the rate of breastfeeding around the world [11,123,124,125].

Fortunately, most of the collected papers (70%) recommended SSC between mother and infant, but in 39%, precautions such as wearing a surgical mask by the mother and hands and surfaces disinfection were proposed to mitigate the risk of transmission of SARS-CoV-2 from the mother to newborn. Recommendations against practicing SSC were slightly more often formulated in the first two quarters of 2020 when concerns about the potential risk of COVID-19 infection outweighed the known benefits of SSC. However, they can be found in recommendations regarding perinatal care submitted on the national level after June 2020, when the WHO stated that SSC contact and breastfeeding should still be encouraged for new mothers and their babies in cases of suspected or confirmed COVID-19 [126] (Supplementary Table S5).

It is important to note that during the analyses, we observed some differentiation in the use of the term SSC. At least in two papers, it was referred to as any proximity and close contact between mother and child in the postpartum period [78,84]. Taking a straightforward understanding of the definition SSC should refer to the practice in the delivery room where a baby is dried and laid directly on the mother’s bare chest after birth, both covered and staying together for at least the first 1–2 h after birth with no interruptions until baby finishes his first feeding [121]. SSC and first breastfeeding helps colonize the newborn with desired maternal microbes, which would not happen if the breasts were washed; this is a reason to exclude such behavior from perinatal recommendation, even for prevention of COVID-19 transmission, as highlighted by Gribble et al. [86]. Physical proximity, calmness, and proper duration of contact are important for the quality of SSC. The use of masks, gloves, and the need for a disinfected environment, proposed in many recommendations in this context, must interfere with SSC (Figure 4). Therefore, some authors highlighted that SSC should be practiced in line with pre-pandemic conditions as part of routine care disregarding maternal COVID-19 status, as the benefits outweigh the potential infection risk [42]. Especially for complicated and preterm deliveries, skin-to-skin practice should also continue during neonatal care in the hospital and at home as kangaroo care with family members other than the mother.

It is interesting to note that only one paper indicated that kangaroo care could be performed by a healthy person other than the COVID-19 infected mother. Father replacement in SSC is often practiced during delivery by cesarean section due to medical considerations other than COVID-19. It is well known that SSC provides many emotional benefits for mother and baby and helps build proximity and bonding between mother and child based on a neuro-hormonal manner. What is more, as important as it is for the mother-child dyad, the shedding of oxytocin during SSC plays a role in father–infant bonding [127]. Considering the importance of SSC in perinatal care and beyond the span live of neonates, several authors of the analyzed recommendations referred to limiting SSC as an emerging problem that may have severe consequences for maternal and infant diminished well-being and breastfeeding rates on the cohort of newborns of COVID-19 infected mothers (see Supplementary Table S5). An important problem raised in 12 papers (out of 56 that referred to this issue) [57,59,63,69,71,72,80,81,82,86,90,101] was that recommendations regarding SSC were very diverse and often conflicting in the time of the COVID-19 pandemic.

The same problem was found with the recommendations related to breastfeeding and the use of human milk. On 13 March 2020, the WHO [17] advised early (within one hour of birth) and exclusive breastfeeding for COVID-19 suspected and confirmed mothers while paying attention to adequate precautions. Although many countries have followed the WHO guidelines, others have taken as an overarching goal to stop the spread of infection by implementing mother–baby separation policies and restricting access to breast milk [101]. At the same time, the CDC does not categorically prohibit breastfeeding but instead pays attention to adequate precautions to avoid spreading the virus from COVID-19 confirmed mothers or symptomatic women to the babies [128].

Surprisingly, as many as 43% of papers counseling on breastfeeding did not provide detailed information about precautions concerning breastfeeding. What is worse, in seven publications (four on the non-country specific level and three national ones) [24,31,34,40,64,77,95], direct breastfeeding was suspended. According to the AAP and WHO recommendations, fresh breast milk is the first option when a baby cannot be put into the breast [129,130].

The nutritional value and biological activity of expressed mother’s own milk (EMM) is also important for the baby’s development, and the process of pumping allows the mother to maintain lactation and return to breastfeeding as soon as possible. Unfortunately, this option was too rarely recommended in the analyzed papers. Only 23 of them (out of 73) allowed the feeding of babies by EMM under a variety of conditions. A relatively common condition for EMM feeding that occurs in six recommendations was the presence of a healthy caregiver who would feed the baby. On the other hand, the involvement of third parties in the process of feeding the newborn can be supportive for COVID-19 infected mothers and bring her relief and reduce stress; otherwise, it is unrealistic in the time of other pandemic restrictions with no visitors in hospitals, social distancing, and shortage of health professionals in the maternity ward. Ten papers indicated this option for asymptomatic mothers (Table 3); seven recognized it as the best way of feeding newborns of mothers who are severely ill or separated because of pandemic restrictions.

While we assume that these policies were intended to protect infants from COVID-19 infection, they may not fully address the long-term consequences of separation and not putting the baby onto the breast just after delivery. Breastfeeding on demand from the first hour after delivery keeps enough mother’s milk supply and addresses a baby’s psychological and emotional needs. Therefore, we recognized recommendations to diminish the frequency of direct breastfeeding to one to two days as unreasonable and damaging for the well-being of mother and child [87].

It is well documented that donor human milk (DHM) is the preferred feeding strategy, especially for preterm newborns and all other infants, when mothers’ own milk is not available [131,132,133]. This recommendation is supported by the evidence concerning lower rates of necrotizing enterocolitis, improved feeding tolerance (in comparison with infant formula), and reduced cardiovascular risk factors in adolescents [134,135]. Unfortunately, only 15 of the studies enclosed in the present review (17%) provided information about human donor milk and milk banking service. During the COVID-19 pandemic, many hospitals have suspended human milk banking services due to the reduced number of personnel, resulting from imposed quarantine or isolation [67]. In Brazil [91], most of the medical centers did not accept milk donation due to fear of SARS-CoV-2 infection (23 out of 24, 95.8% of reviewed hospitals). Therefore, as it was reported by the Collaborative Network of Milk Banks and Associations, the significant decrease in human milk donation rates was globally observed in the time of the COVID-19 pandemic. Data collected last year estimate that 500,000 infants born worldwide below 32 gestation weeks lacked access to donor milk which has increased in demand due to perinatal changes in the time of the COVID-19 pandemic [136]. When expressed mothers’ own milk was not available, DHM was indicated as the best form of infant feeding. However, Asadi et al. [40] indicated that in case of a mother’s severe illness, the infant should be fed with human milk (stored in a milk bank) or formula, suggesting that both feeding patterns are equal, which is not scientifically proven [134]. Considering that DHM improves the immediate and long-term health and well-being of the individual infant, its role and importance should always be highlighted when EMM is not available, including mothers critically ill with COVID-19.

The present scoping review has several weaknesses that make it likely that the picture on COVID-19 perinatal care is not comprehensive. Our query was limited to the texts published in English; therefore, the insight into national regulations is not complete. It should be noted, however, that most of the national-level texts come from non-English speaking countries. Additionally, recommendations published by international and national societies or institutions on their websites/newsletters might not be included in the researched databases if not quoted in scientific publications. The strength of our review is that it was conducted by a team consisting of medical and social scientists, representatives of parent organizations promoting breastfeeding, and hospital staff, which allowed interdisciplinary coverage of the topic of maternal and child well-being in context to support breastfeeding.

## 5. Conclusions

Breastfeeding is one aspect of a woman’s maternal identity, so hospital practices that support it have great implications for maternal and child well-being. In analyzing COVID-19 recommendations, we noted the recommendation to allow and protect breastfeeding of mothers infected with SARS-CoV-2, as being raised most often by global and national authorities, despite very limited initial knowledge of the risk of transmitting the SARS-CoV-2 through this route from mother to child. However, discrepancies in specific guidelines concerning good practices supporting breastfeeding (SSC, rooming-in, natural delivery, donor milk provision) do not reflect the overarching goal. The lack of coherent, evidence-based recommendations and often conflicting character of guidelines issued by well-established authorities was a problem discussed by the authors of the recommendations. Moreover, we also observed a great differentiation in analyzed recommendations. In the situation of a global threat such as the COVID-19 pandemic, it seems important to create a consistent message in order not to create chaos. No consideration was given to the fact that, in the absence of sufficient knowledge about the risk of infant SARS-CoV-2 infection, to enable evidence-based decision-making about SSC, rooming-in or separation, and mode of feeding, a directive decision without respect for the mother’s wishes may have the most negative impact on her and her child’s well-being. Only a limited number of recommendations referred to shared decision-making and informed choice. Moreover, recommendations rarely recommend a decision-making process based on clinical conditions, which is alarming.

## Figures and Tables

**Table 1:** Inclusion and exclusion criteria.

Area	Inclusion Criteria	Exclusion Criteria
	Proposes own recommendations or reaffirms third-party ones	Summarizes research/study but neither proposes nor reaffirms any recommendations
Type of publication	Systematic review, meta-analysis, review of recommendations narrative, scoping, rapid review original paper	Case reports, letters, commentaries, editorials, short reports, records of pregnancy during COVID19
Researched population	Multi-center, all-population studies, national-level studies, international studies	One-center studies, local (one district, one city) studies, case studies
Key issues in the text	Obstetric, management in pregnancy, perinatal care, safety breastfeeding during COVID-19, breastfeeding support, mitigate the risk the infection from mothers to babies	COVID-19 outcomes of the mothers and babies, vaccination safety during breastfeeding, IgG, IgM, seroconversion, epidemiology of COVID-19 in women and neonates, clinical characteristics of COVID-19 in neonates and pregnant women, diagnosis, and therapy of COVID-19
Language	English	Other

**Table 2:** Allocation of the recommendations.

Localization	Number of Papers
Non-country specific	45
Argentina	1
Australia	2
Brazil	5
China	1
Egypt	1
France	1
India	3
Italy	7
Japan	1
Nigeria	2
Poland	2
Russia	1
Saudi Arabia	1
Southeast Asia	1
Spain	3
Turkey	1
UK	2
USA	6

**Table 3:** Recommendations concerning breastfeeding for mothers with confirmed and/or suspected COVID-19.

Author and Date of Publication	Direct BF ^1^ Is Recommended	in Confirmed Mothers Direct BF Is Not Recommended	Direct BF Recommended with Certain Precautions	Feeding EMM ^2^ by a Healthy Caregiver	Feeding EMM by Asymptomatic Mother	Feeding EMM If the Mother Is Severely Ill or Temporary Separation Is Needed	DHM ^3^ from Healthy Mother Is Recommended When EMM Is Not Available	Infant Formula When Mother Is Confirmed
Liang, March 2020 [95]		+		+				
WHO, April 2020 [17]	+						+	
CalilVMLT, April 2020 [62]	+				+		+	
Asadi, April 2020 [40]		+					+	+
Donders, April 2020 [23]			+		+			
Narang, May 2020 [36]			+					
Williams, May 2020 [88]			+				+	
Tomori, May 2020 [76]	+							
Abdollahpour, May 2020 [24]		+					+	
Pramana, June 2020 [77]		+						
TrapaniJúnior, June 2020 [25]			+	+				
ShahbaziSighaldeh, June 2020 [63]	+				+			
Trevisanuto, June 2020 [60]			+					
Lavizzari, June 2020 [55]			+					
Goyal, July 2020 [38]			+			+		
Api, July 2020 [50]			+					
Choi, August 2020 [78]	+							
Ryan, August 2020 [98]	+				+			
Davanzo, August 2020 [69]	+							
Mascarenhas, August 2020 [26]	+					+		
Mocelin, September 2020 [99]								
NgYPM, September 2020 [79]	+				+		+	
Genoni, September 2020 [80]	+							
Krupa, September 2020 [49]	+							
Góes, October 2020 [28]	+							
Benski, November 2020 [100]			+					
Dimopoulou, November 2020 [81]	+							
Yeo, November 2020 [92]			+					
VuHoang, December 2020 [101]			+					
Haiek, January 2021 [70]			+		+		+	+
Kotlar, January 2021 [59]	+							
Rollins, February 2021 [102]	+							
Spatz, February 2021 [82]	+							
Bartick, March 2021 [83]								
Olonan-Jusi, March 2021 [89]	+				+		+	
vanVeenendaal, March 2021 [65]	+							
Vassilopoulou, April 2021 [103]	+							
Yeo, April 2021 [71]			+				+	+
Pountoukidou, April 2021 [74]			+					
	Australia							
Vogel, December 2020 [18]			+					
	Brazil							
deCarvalho May 2020 [68]	+							
Stofel August 2020 [51]			+					
deOliveira February 2021 [29]			+				+	
Cardoso February 2021 [30]			+					
Gonçalves-Ferri March 2021 [91]	+							
	China							
Chen, March 2020 [31]		+						
	Egypt							
Mostafa, August 2020 [66]			+					+
	France							
Vivanti, May 2020 [104]			+					
	India							
Chawla, June 2020 [61]	+			+				
Sachdeva, June 2020 [67]			+		+		+	
Sharma, August 2020 [32]			+			+		
Italy								
Davanzo, March 2020 [97]	+					+		
Franchi, March 2020 [41]			+					
Moro, November 2020 [96]			+				+	
Singh, November 2020 [33]			+			+		
Ronchi 12, 2020 [93]			+					
Giusti, April 2021 [42]	+					+	+	
Nigeria								
Ezenwa, May 2020 [87]	+							+
Okunade, July 2020 [43]			+			+		
Poland								
Kalinka, January 2021 [44]	+							
Wszolek, April 2021 [53]			+					
Russia								
Ignatko, May 2020 [45]			+					
Saudi Arabia								
Faden, August 2020 [34]		+		+				+
Spain								
López, June 2020 [54]			+		+			
Montes, July 2020 [75]	+				+		+	
LalagunaMallada, July 2020 [85]			+					
Turkey								
Erdeve, June 2020 [46]			+		+			
United Kingdom								
Ross-Davie, March 2021 [47]			+					
United States								
Boelig, May 2020 [58]			+					
Amatya, May 2020 [64]		+		+			+	
Harriel, August 2020 [84]			+	+				
Boelig, October 2020 [48]			+					
Flannery, April 2021 [72]	+							

^1^ BF—Breastfeeding, ^2^ EMM—own mother’s milk,^3^ DHM—donor human milk.

## Data Availability

Not applicable.

## Supplementary Materials

The following are available online at https://www.mdpi.com/article/10.3390/ijerph19063347/s1, Table S1: Search strategy; Table S2: List of studies included; Table S3: Recommendations concerning the mode of delivery and companions on the labor for mothers with confirmed and/or suspected COVID-19; Table S4: Recommendations concerning visitor policies on maternity ward for mothers with confirmed and/or suspected COVID-19; Table S5: Recommendations concerning skin to skin contact for mothers with confirmed and/or suspected COVID-19; Table S6: Recommendations concerning rooming-in management for mothers with confirmed and/or suspected COVID-19.
