# Supplementary material for: Protecting Breastfeeding during the COVID-19 Pandemic: A Scoping Review of Perinatal Care Recommendations in the Context of Maternal and Child Well-Being
Source: Int J Environ Res Public Health. 2022 Mar 11;19(6):3347. doi: 10.3390/ijerph19063347 (PMC8949921; doi:10.3390/ijerph19063347)
Supplement: Supplementary file 1 [file ijerph-19-03347-s001.zip › Suplementary Table S1 search strategy.pdf]

Supplementary Table S1. Search strategy.

	(A)	AND (B)	AND (C)	Timespan	Notes
Web of Science Core Collection	“delivery” OR “labor” OR “labour” OR “giving birth” OR “breastmilk” OR “human milk” OR “donor milk” OR “own mothers milk” OR “breastfeeding” OR “mother – infant contact” OR “skin to skin” OR “prenatal care” OR “postnatal care” OR “perinatal care”	“COVID-19” OR “COVID19” OR “coronavirus” OR “SARS-CoV-2”	“recommendation” OR “counseling” OR “policy“ OR “guidelines” OR “guidance” OR “statement” OR “best practices”	Timespan: 2020-03-01 to 2021-05-31 (Publication Date)	Each search string All=(“A” AND “B” AND “C”) was entered separately
Medline via PubMed	“delivery” OR “labor” OR “labour” OR “giving birth” OR “breastmilk” OR “human milk” OR “donor milk” OR “own mothers milk” OR “breastfeeding” OR “mother – infant contact” OR “skin to skin” OR “prenatal care” OR “postnatal care” OR “perinatal care”	“COVID-19” OR “COVID19” OR “coronavirus” OR “SARS-CoV-2”	“recommendation” OR “counseling” OR “policy“ OR “guidelines” OR “guidance” OR “statement” OR “best practices”	(2020/3/1:2021/5/31[pdat])	Each search string ("A"[All Fields] AND "B"[All Fields] AND "C"[All Fields]) AND (2020/3/1:2021/5/31[pdat]) was entered separately

Scopus	“delivery” OR “labor” OR “labour” OR “giving birth” OR “breastmilk” OR “human milk” OR “donor milk” OR “own mothers milk” OR “breastfeeding” OR “mother – infant contact” OR “skin to skin” OR “prenatal care” OR “postnatal care” OR “perinatal care”	“COVID-19” OR “COVID19” OR “coronavirus” OR “SARS-CoV-2”	“recommendation” OR “counseling” OR “policy“ OR “guidelines” OR “guidance” OR “statement” OR “best practices”	PUBYEAR > 2019 AND PUBYEAR < 2022	Each search string (TITLE-ABS-KEY (“A” AND “B” AND “C”) AND PUBYEAR > 2019 AND PUBYEAR < 2022 was entered separately Publications earlier than 1 of March 2020 and later than 31 of May 2021 were manually excluded from the export
