# Supplementary material for: Protecting Breastfeeding during the COVID-19 Pandemic: A Scoping Review of Perinatal Care Recommendations in the Context of Maternal and Child Well-Being
Source: Int J Environ Res Public Health. 2022 Mar 11;19(6):3347. doi: 10.3390/ijerph19063347 (PMC8949921; doi:10.3390/ijerph19063347)
Supplement: Supplementary file 1 [file ijerph-19-03347-s001.zip › Supplementary Table S2 list of studies.pdf]

Supplementary Table S2. List of studies included.

Autor(s)	publication date	Title	country/global level	type of publication
Chen, D.; Yang, H.; Cao, Y.; Cheng, W.; Duan, T.; Fan, C.; Fan, S.; Feng, L.; Gao, Y.; He, F.; He, J.; Hu, Y.; Jiang, Y.; Li, Y.; Li, J.; Li, X.; Lin, K.; Liu, C.; Liu, J.; Liu, X.; Pan, X.; Pang, Q.; Pu, M.; Qi, H.; Shi, C.; Sun, Y.; Sun, J.; Wang, X.; Wang, Y.; Wang, Z.; Wang, C.; Wu, S.; Xin, H.; Yan, J.; Zhao, Y.; Zheng, J.; Zhou, Y.; Zou, L.; Zeng, Y.; Zhang, Y.; Guan, X.	03-2020	Expert consensus for managing pregnant women and neonates born to mothers with suspected or confirmed novel coronavirus (COVID-19) infection	China, USA	review of recommendations
Davanzo, R.; Merewood, A.; Manzoni, P.	03-2020	Breastfeeding and coronavirus disease-2019: Ad interim indications of the Italian Society of Neonatology endorsed by the Union of European Neonatal & Perinatal Societies	Italy	review of recommendations
Franchi, M.; Bosco, M.; Garzon, S.; Laganà, A.S.; Cromi, A.; Barbieri, B.; Raffaelli, R.; Tacconelli, E.; Scambia, G.; Ghezzi, F.	03-2020	Management of obstetrics and gynaecological patients with COVID-19	Italy	review of recommendations
Liang, H.; Acharya, G.	03-2020	Novel corona virus disease (COVID-19) in pregnancy: What clinical recommendations to follow?	global spectrum	a query of research
Asadi, L.; Tabatabaei, R.S.; Safinejad, H.; Mohammadi, M.	04-2020	New corona virus (COVID-19) management in pregnancy and childbirth	global spectrum	systematic review
Calil VMLT; Krebs VLJ; Carvalho WB	04-2020	Guidance on breastfeeding during the Covid-19 pandemic.	global spectrum	a query of research
Donders, F.; Lonnée-Hoffmann, R.; Tsiakalos, A.; Mendling, W.; De Oliveira, J.M.; Judlin, P.; Xue, F.; Donders, G.G.G.	04-2020	ISIDOG recommendations concerning COVID-19 and pregnancy	global spectrum	a query of research
Poon LC; Yang H; Kapur A; Melamed N; Dao B; Divakar H; McIntyre HD; Kihara AB; Ayres-de-Campos D; Ferrazzi EM; Di Renzo GC; Hod M	04-2020	Global interim guidance on coronavirus disease 2019 (COVID-19) during pregnancy and puerperium from	global spectrum	a query of research

		FIGO and allied partners: Information for healthcare professionals.		
Stephens, A.J.; Barton, J.R.; Bentum, N.-A.A.; Blackwell, S.C.; Sibai, B.M.	04-2020	General Guidelines in the Management of an Obstetrical Patient on the Labor and Delivery Unit during the COVID-19 Pandemic	global spectrum	a query of research
WHO	04-2020	WHO Frequently Asked Questions : Breastfeeding and COVID-19 For health care workers.	global spectrum	review of recommendations
Abdollahpour, S.; Khadivzadeh, T.	05-2020	Improving the quality of care in pregnancy and childbirth with coronavirus (COVID-19): a systematic review	global spectrum	systematic review
Amatya, S.; Corr, T.E.; Gandhi, C.K.; Glass, K.M.; Kresch, M.J.; Mujsce, D.J.; Oji-Mmuo, C.N.; Mola, S.J.; Murray, Y.L.; Palmer, T.W.; Singh, M.; Fricchione, A.; Arnold, J.; Prentice, D.; Bridgeman, C.R.; Smith, B.M.; Gavigan, P.J.; Ericson, J.E.; Miller, J.R.; Pauli, J.M.; Williams, D.C.; McSherry, G.D.; Legro, R.S.; Iriana, S.M.; Kaiser, J.R.	05-2020	Management of newborns exposed to mothers with confirmed or suspected COVID-19	USA	a query of research
BMJ	05-2020	Covid-19 and pregnancy.	UK	review of recommendations
Boelig, R.C.; Lambert, C.; Pena, J.A.; Stone, J.; Bernstein, P.S.; Berghella, V.	05-2020	Labor and delivery guidance for COVID-19	USA	review of recommendations
de Carvalho, W.B.; Gibelli, M.A.B.C.; Krebs, V.L.J.; Tragante, C.R.; Perondi, M.B.M.	05-2020	Role of a neonatal intensive care unit during the COVID-19 pandemia: Recommendations from the neonatology discipline	Brazil	review of recommendations
Ezenwa, B.N.; Fajolu, I.B.; Akinajo, O.R.; Makwe, C.C.; Oluwole, A.A.; Akase, I.E.; Afolabi, B.B.; Ezeaka, V.C.	05-2020	Management of covid-19: a practical guideline for maternal and newborn health care providers in Sub-Saharan Africa	Nigeria	a query of research
Ignatko, I.V.; Strizhakov, A.N.; Timokhina, E.V.; Denisova, Y.V.	05-2020	Novel coronavirus infection (Covid-19): Guiding principles for obstetric care under pandemic conditions	Russia	a query of research
Narang K; Ibirogba ER; Elrefaei A; Trad ATA; Theiler R; Nomura R; Picone O; Kilby M; Escuriet R; Suy A; Carreras E; Tonni G; Ruano R	05-2020	SARS-CoV-2 in Pregnancy: A Comprehensive Summary of Current Guidelines.	global spectrum	review of recommendations

Tomori C; Gribble K; Palmquist AEL; Ververs MT; Gross MS	05-2020	When separation is not the answer: Breastfeeding mothers and infants affected by COVID-19.	global spectrum	a query of research
Vivanti AJ; Deruelle P; Picone O; Guillaume S; Roze JC; Mulin B; Kochert F; De Beco I; Mahut S; Gantois A; Barasinski C; Petitprez K; Pauchet-Traversat AF; Droy A; Benachi A	05-2020	Post-natal follow-up for women and neonates during the COVID-19 pandemic: French National Authority for Health recommendations.	France	review of recommendations
Williams J; Namazova-Baranova L; Weber M; Vural M; Mestrovic J; Carrasco-Sanz A; Breda J; Berdzuli N; Pettoello-Mantovani M	05-2020	The Importance of Continuing Breastfeeding during Coronavirus Disease-2019: In Support of the World Health Organization Statement on Breastfeeding during the Pandemic.	global spectrum	a query of research
Chawla D; Chirla D; Dalwai S; Deorari AK; Ganatra A; Gandhi A; Kabra NS; Kumar P; Mittal P; Parekh BJ; Sankar MJ; Singhal T; Sivanandan S; Tank P	06-2020	Perinatal-Neonatal Management of COVID-19 Infection - Guidelines of the Federation of Obstetric and Gynaecological Societies of India (FOGSI), National Neonatology Forum of India (NNF), and Indian Academy of Pediatrics (IAP).	India	rapid/living/scoping review
Erdeve, Ö.; Çetinkaya, M.; Baş, A.Y.; Narlı, N.; Duman, N.; Vural, M.; Koç, E.	06-2020	The Turkish neonatal society proposal for the management of COVID-19 in the neonatal intensive care unit	Turkey	a query of research
Lavizzari, A.; Klingenberg, C.; Profit, J.; Zupancic, J.A.F.; Davis, A.S.; Mosca, F.; Molloy, E.J.; Roehr, C.C.; Bassler, D.; Burn-Murdoch, J.; Danhaive, O.; Davis, J.; Ferri, W.A.G.; Fuchs, H.; Ge, H.; Gupta, A.; Gupta, M.; Lang, A.; van Kaam, A.; Díaz, V.J.L.; Treviño-Pérez, R.; Helkey, D.; Tembulkar, S.; Mariani, G.L.; Naver, L.; Patel, A.; Shah, P.; Szczapa, T.; Vento, M.; Wellmann, S.; Zangen, S.	06-2020	International comparison of guidelines for managing neonates at the early phase of the SARS-CoV-2 pandemic	global spectrum	original paper
López, M.; Gonce, A.; Meler, E.; Plaza, A.; Hernández, S.; Martinez-Portilla, R.J.; Cobo, T.; García, F.; Gómez Roig, M.D.; Gratacós, E.; Palacio, M.; Figueras, F.	06-2020	Coronavirus Disease 2019 in Pregnancy: A Clinical Management Protocol and Considerations for Practice	Spain	a query of research
Pramana, C.; Suwantoro, J.; Sumarni, N.; Kumalasari, M.L.F.; Selasih Putri Isnawati, H.;	06-2020	Breastfeeding in postpartum women infected with COVID-19	global spectrum	review of recommendations

Supinganto, A.; Ernawati, K.; Sirait, L.I.; Staryo, N.; Nurhidayah; Dwiyono, K.				
Shahbazi Sighaldeh, S.; Ebrahimi Kalan, M.	06-2020	Care of newborns born to mothers with COVID-19 infection; a review of existing evidence	global spectrum	a query of research
Trapani Júnior, A.; Vanhoni, L.R.; Silveira, S.K.; Marcolin, A.C.	06-2020	Childbirth, Puerperium and Abortion Care Protocol during the COVID-19 Pandemic	global spectrum	a query of research
Trevisanuto, D.; Weiner, G.; Lakshminrusimha, S.; Azzimonti, G.; Nsubuga, J.B.; Velaphi, S.; Seni, A.H.A.; Tylleskär, T.; Putoto, G.	06-2020	Management of mothers and neonates in low resources setting during covid-19 pandemia	global spectrum	a query of research
Api, O.; Sen, C.; Debska, M.; Saccone, G.; D'Antonio, F.; Volpe, N.; Yayla, M.; Esin, S.; Turan, S.; Kurjak, A.; Chervenak, F.	07-2020	Clinical management of coronavirus disease 2019 (COVID-19) in pregnancy: Recommendations of WAPM-World Association of Perinatal Medicine	global spectrum	a query of research
Ashokka, B.; Loh, M.-H.; Tan, C.H.; Su, L.L.; Young, B.E.; Lye, D.C.; Biswas, A.; Illanes, S.E.; Choolani, M.	07-2020	Care of the pregnant woman with coronavirus disease 2019 in labor and delivery: anesthesia, emergency cesarean delivery, differential diagnosis in the acutely ill parturient, care of the newborn, and protection of the healthcare personnel	global spectrum	a query of research
Goyal, M.; Singh, P.; Melana, N.	07-2020	Review of care and management of pregnant women during COVID-19 pandemic	global spectrum	a query of research
Lalaguna Mallada P; Díaz-Gómez NM; Costa Romero M; San Feliciano Martín L; Gabarrell Guiu C	07-2020	[The impact of Covid-19 pandemic on breastfeeding and birth care. The importance of recovering good practices.].	Spain	a query of research
Montes, M.T.; Herranz-Rubia, N.; Ferrero, A.; Flórez, A.; Quiroga, A.; Gómez, A.; Chinea, B.; Gómez, C.; Montaner, C.; Sánchez, N.; Rico, C.M.; Segovia, C.; Eiriz, D.; Carrillo, E.; Cañizares, E.M.; Chattas, G.; Mimón, I.I.; Guerra, I.M.; Del Río, L.; Martín, M.J.; Elena, M.O.; García, M.; Alcázar, M.; Martínez, P.; Sánchez, P.; De Miguel, R.; Cortés, R.; Massip, S.; Tato, S.; Jiménez, T.	07-2020	Neonatal nursing in the COVID-19 pandemic: can we improve the future?	Spain	review of recommendations
Okunade, K.S.; Makwe, C.C.; Akinajo, O.R.; Owie, E.; Ohazurike, E.O.; Babah, O.A.; Okunowo, A.A.;	07-2020	Good clinical practice advice for the management of pregnant women with suspected or confirmed COVID-19 in Nigeria	Nigeria	review of recommendations

Omisakin, S.I.; Oluwole, A.A.; Olamijulo, J.A.; Adegbola, O.; Anorlu, R.I.; Afolabi, B.B.				
Choi KR; Records K; Low LK; Alhusen JL; Kenner C; Bloch JR; Premji SS; Hannan J; Anderson CM; Yeo S; Cynthia Logsdon M	08-2020	Promotion of Maternal-Infant Mental Health and Trauma-Informed Care During the COVID-19 Pandemic.	global spectrum	review of recommendations
Davanzo, R.; Moro, G.; Sandri, F.; Agosti, M.; Moretti, C.; Mosca, F.	08-2020	Skin-to-Skin Contact at Birth in the COVID-19 Era: In Need of Help!	global spectrum	a query of research
Faden YA; Alghilan NA; Alawami SH; Alsulmi ES; Alsum HA; Katib YA; Sabr YS; Tahir FH; Bondagji NS	08-2020	Saudi Society of Maternal-Fetal Medicine guidance on pregnancy and coronavirus disease 2019.	Saudi Arabia	a query of research
Harriel, K.L.; Nolt, D.; Moore, S.; Kressly, S.; Bernstein, H.H.	08-2020	Management of neonates after postpartum discharge and all children in the ambulatory setting during the coronavirus disease 2019 (COVID-19) pandemic	USA	a query of research
Mascarenhas, V.H.A.; Caroci-Becker, A.; Venâncio, K.C.M.P.; Baraldi, N.G.; Durkin, A.C.; Riesco, M.L.G.	08-2020	Care recommendations for parturient and postpartum women and newborns during the covid-19 pandemic: A scoping review	global spectrum	rapid/living/scoping review
Mostafa, A. S.; Abdalbaky, A.; Fouda, E. M.; Shaaban, H. H.; Elnady, H. G.; Hassab-Allah, M.; Rashad, M. M.; El Attar, M. M.; Alfishawy, M.; Hussien, S. M.; Hamed, T.; Hamed, D. H.; Sarhan, D. T.	08-2020	Practical approach to COVID-19: an Egyptian pediatric consensus	Egypt	review of recommendations
Ryan GA; Purandare NC; McAuliffe FM; Hod M; Purandare CN	08-2020	Clinical update on COVID-19 in pregnancy: A review article.	global spectrum	a query of research
Sachdeva RC; Jain S; Mukherjee S; Singh J	08-2020	Ensuring Exclusive Human Milk Diet for All Babies in COVID-19 Times.	India	a query of research
Sharma, J.B.; Sharma, E.; Sharma, S.; Singh, J.	08-2020	Recommendations for prenatal, intrapartum, and postpartum care during COVID-19 pandemic in India	India	a query of research
Stofel, N.S.; Christinelli, D.; Silva, R.C.S.; Salim, N.R.; Beleza, A.C.S.; Bussadori, J.C.C.	08-2020	Perinatal care in the COVID-19 pandemic: Analysis of Brazilian guidelines and protocols	Brazil	review of recommendations
Czeresnia RM; Trad ATA; Britto ISW; Negrini R; Nomura ML; Pires P; Costa FDS; Nomura RMY; Ruano R	09-2020	SARS-CoV-2 and Pregnancy: A Review of the Facts.	global spectrum	a query of research

Genoni G; Conio A; Binotti M; Manzoni P; Castagno M; Rabbone I; Monzani A	09-2020	Management and Nutrition of Neonates during the COVID-19 Pandemic: A Review of the Existing Guidelines and Recommendations.	global spectrum	rapid/living/scoping review
Krupa, A.; Schmidt, M.; Zborowska, K.; Jorg, D.; Czajkowska, M.; Skrzypulec-Plinta, V.	09-2020	Impact of COVID-19 on pregnancy and delivery - Current knowledge	global spectrum	a query of research
Mocelin, H.J.S.; Primo, C.C.; Laignier, M.R.	09-2020	Overview on the recommendations for breastfeeding and COVID-19	global spectrum	rapid/living/scoping review
Ng YPM; Low YF; Goh XL; Fok D; Amin Z	09-2020	Breastfeeding in COVID-19: A Pragmatic Approach.	global spectrum	a query of research
Boelig, R.C.; Manuck, T.; Oliver, E.A.; Di Mascio, D.; Saccone, G.; Bellussi, F.; Berghella, V.	10-2020	Obstetric protocols in the setting of a pandemic	USA	a query of research
Góes, F.G.B.; Dos Santos, A.S.T.; Lucchese, I.; da Silva, L.J.; da Silva, L.F.; Silva, M.A.	10-2020	Best practices in newborn care in COVID-19 times: An integrative review	global spectrum	systematic review
Benski C; Di Filippo D; Taraschi G; Reich MR	11-2020	Guidelines for Pregnancy Management During the COVID-19 Pandemic: A Public Health Conundrum.	global spectrum	review of recommendations
Dimopoulou D; Triantafyllidou P; Daskalaki A; Syridou G; Papaevangelou V	11-2020	Breastfeeding during the novel coronavirus (COVID-19) pandemic: guidelines and challenges.	global spectrum	a query of research
Gribble K; Marinelli KA; Tomori C; Gross MS	11-2020	Implications of the COVID-19 Pandemic Response for Breastfeeding, Maternal Caregiving Capacity and Infant Mental Health.	Australia	review of recommendations
Moro GE; Bertino E	11-2020	Breastfeeding, Human Milk Collection and Containers, and Human Milk Banking: Hot Topics During the COVID-19 Pandemic.	Italy	a query of research
Perrine CG; Chiang KV; Anstey EH; Grossniklaus DA; Boundy EO; Sauber-Schatz EK; Nelson JM	11-2020	Implementation of Hospital Practices Supportive of Breastfeeding in the Context of COVID-19 - United States, July 15-August 20, 2020.	USA	original paper
Singh V; Trigunait P; Majumdar S; Ganeshan R; Sahu R	11-2020	Managing pregnancy in COVID-19 pandemic: A review article.	Italy	a query of research
Yeo, K.T.; Oei, J.L.; De Luca, D.; Schmölzer, G.M.; Guaran, R.; Palasanthiran, P.; Kumar, K.; Buonocore, G.; Cheong, J.; Owen, L.S.; Kusuda, S.; James, J.; Lim, G.; Sharma, A.; Uthaya, S.; Gale, C.; Whittaker, E.; Battersby, C.; Modi, N.; Norman, M.;	11-2020	Review of guidelines and recommendations from 17 countries highlights the challenges that clinicians face caring for neonates born to mothers with COVID-19	global spectrum	a query of research

Naver, L.; Giannoni, E.; Diambomba, Y.; Shah, P.S.; Gagliardi, L.; Harrison, M.; Pillay, S.; Alburaey, A.; Yuan, Y.; Zhang, H.				
Barrero-Castillero A; Beam KS; Bernardini LB; Ramos EGC; Davenport PE; Duncan AR; Fraiman YS; Frazer LC; Healy H; Herzberg EM; Keyes ML; Leeman KT; Leone K; Levin JC; Lin M; Raju RM; Sullivan A	12-2020	COVID-19: neonatal-perinatal perspectives.	global spectrum	a query of research
Ronchi A; Pietrasanta C; Zavattoni M; Saruggia M; Schena F; Sinelli MT; Agosti M; Tzialla C; Varsalone FF; Testa L; Ballerini C; Ferrari S; Mangili G; Ventura ML; Perniciaro S; Spada E; Lunghi G; Piralla A; Baldanti F; Mosca F; Pugni L	12-2020	Evaluation of Rooming-in Practice for Neonates Born to Mothers With Severe Acute Respiratory Syndrome Coronavirus 2 Infection in Italy.	Italy	original paper
Vogel JP; Tendal B; Giles M; Whitehead C; Burton W; Chakraborty S; Cheyne S; Downton T; Fraile Navarro D; Gleeson G; Gordon A; Hunt J; Kitschke J; McDonald S; McDonnell N; Middleton P; Millard T; Murano M; Oats J; Tate R; White H; Elliott J; Roach V; Homer CSE	12-2020	Clinical care of pregnant and postpartum women with COVID-19: Living recommendations from the National COVID-19 Clinical Evidence Taskforce.	Australia	rapid/living/scoping review
Vu Hoang D; Cashin J; Gribble K; Marinelli K; Mathisen R	12-2020	Misalignment of global COVID-19 breastfeeding and newborn care guidelines with World Health Organization recommendations.	global spectrum	review of recommendations
Haiek LN; LeDrew M; Charette C; Bartick M	01-2021	Shared decision-making for infant feeding and care during the coronavirus disease 2019 pandemic.	global spectrum	a query of research
Kalinka J; Wielgos M; Leszczynska-Gorzelak B; Piekarska A; Huras H; Sieroszewski P; Czajkowski K; Wysocki J; Lauterbach R; Helwich E; Mazela J	01-2021	COVID-19 impact on perinatal care: risk factors, clinical manifestation and prophylaxis. Polish experts' opinion for December 2020.	Poland	a query of research
Kotlar, B.; Gerson, E.; Petrillo, S.; Langer, A.; Tiemeier, H.	01-2021	The impact of the COVID-19 pandemic on maternal and perinatal health: a scoping review	global spectrum	rapid/living/scoping review
Cardoso, P.C.; de Sousa, T.M.; Rocha, D.D.S.; de Menezes, L.R.D.; Dos Santos, L.C.	02-2021	Maternal and child health in the context of COVID-19 pandemic: evidence, recommendations and challenges	Brazil	a query of research

de Oliveira, M.A.; Silva, N.É.F.; Pereira, J.C.N.; da Silva, S.L.; Caminha, M.F.C.; de Paula, W.K.A.S.; Quirino, G.D.S.; de Oliveira, D.R.; Cruz, R.S.B.L.C.	02-2021	Recommendations for perinatal care in the context of the COVID-19 pandemic	Brazil	a query of research
Rollins, N.; Minckas, N.; Jehan, F.; Lodha, R.; Raiten, D.; Thorne, C.; Van de Perre, P.; Ververs, M.; Walker, N.; Bahl, R.; Victora, C. G.; Chil, Who Covid-Maternal Newborn; Newborn and Infant Feeding, Working	02-2021	A public health approach for deciding policy on infant feeding and mother & ndash;infant contact in the context of COVID-19	global spectrum	a query of research
Spatz, D.L.; Davanzo, R.; Müller, J.A.; Powell, R.; Rigourd, V.; Yates, A.; Geddes, D.T.; van Goudoever, J.B.; Bode, L.	02-2021	Promoting and Protecting Human Milk and Breastfeeding in a COVID-19 World	global spectrum	a query of research
Tolu, L.B.; Feyissa, G.T.; Jeldu, W.G.	02-2021	Guidelines and best practice recommendations on reproductive health services provision amid COVID-19 pandemic: scoping review	global spectrum	rapid/living/scoping review
Bartick MC; Valdés V; Giusti A; Chapin EM; Bhana NB; Hernández-Aguilar MT; Duarte ED; Jenkins L; Gaughan J; Feldman-Winter L	03-2021	Maternal and Infant Outcomes Associated with Maternity Practices Related to COVID-19: The COVID Mothers Study.	global spectrum	original paper
Gonçalves-Ferri, W.A.; Pereira-Cellini, F.M.; Coca, K.; Aragon, D.C.; Nader, P.; Lyra, J.C.; do Vale, M.S.; Marba, S.; Araujo, K.; Dias, L.A.; de Lima Mota Ferreira, D.M.; Nieto, G.; Anchieta, L.M.; de Cássia Silveira, R.; de Moura, M.D.R.; Tuma Calil, V.M.L.; Moraes, V.C.C.; de Almeida, J.H.C.L.; Magalhães, M.; Sonini, T.C.B.; Javorsky, J.B.; Ribeiro, É.L.A.; Ferreira, R.; de Almeida, L.D.C.; Garbers, R.; da Silva Faria, G.M.; Roosch, A.; de Mesquita, A.R.A.; de Oliveira Pinto, R.M.	03-2021	The impact of coronavirus outbreak on breastfeeding guidelines among Brazilian hospitals and maternity services: a cross-sectional study	Brazil	original paper
Olonan-Jusi E; Zambrano PG; Duong VH; Anh NTT; Aye NSS; Chua MC; Kurniasari H; Moe ZW; Ngerncham S; Phuong NTT; Datu-Sanguyo J	03-2021	Human milk banks in the response to COVID-19: a statement of the regional human milk bank network for Southeast Asia and beyond.	Southeast Asia	review of recommendations
van Veenendaal NR; Deierl A; Bacchini F; O'Brien K; Franck LS	03-2021	Supporting parents as essential care partners in neonatal units during the SARS-CoV-2 pandemic.	global spectrum	systematic review

Cavicchiolo, M.E.; Trevisanuto, D.; Priante, E.; Moschino, L.; Mosca, F.; Baraldi, E.	04-2021	Italian neonatologists and SARS-CoV-2: lessons learned to face coming new waves	Italy	systematic review
Flannery, D.D.; Puopolo, K.M.	04-2021	Perinatal COVID-19: guideline development, implementation, and challenges	USA	review of recommendations
Geffner SC; Ávila AS; Etcharrán ML; Fernández AL; Mariani GL; Vain NE	04-2021	Preparedness strategies in neonatology units during the COVID-19 pandemic: A survey conducted at maternity centers in Argentina.	Argentina	original paper
Giusti A; Zambri F; Marchetti F; Corsi E; Preziosi J; Sampaolo L; Pizzi E; Taruscio D; Salerno P; Chiantera A; Colacurci N; Davanzo R; Mosca F; Petrini F; Ramenghi L; Vicario M; Villani A; Viora E; Zanetto F; Chapin EM; Donati S	04-2021	COVID-19 and pregnancy, childbirth, and breastfeeding: the interim guidance of the Italian National Institute of Health.	Italy	review of recommendations
Hosono, S.; Isayama, T.; Sugiura, T.; Kusakawa, I.; Kamei, Y.; Ibara, S.; Tamura, M.; Ishikawa, G.; Enomoto, K.; Okuda, M.; Tanaka, H.; Masaoka, N.; Arahori, H.; Kubo, M.; Shimaoka, H.; Wada, M.	04-2021	Management of infants born to mothers with suspected or confirmed SARS-CoV-2 infection in the delivery room: A tentative proposal 2020	Japan	review of recommendations
Pountoukidou, A.; Potamiti-Komi, M.; Sarri, V.; Papapanou, M.; Routsi, E.; Tsiatsiani, A.M.; Vlahos, N.; Siristatidis, C.	04-2021	Management and prevention of COVID-19 in pregnancy and pandemic obstetric care: A review of current practices	global spectrum	rapid/living/scoping review
Vassilopoulou E; Feketea G; Koumbi L; Mesiari C; Berghea EC; Konstantinou GN	04-2021	Breastfeeding and COVID-19: From Nutrition to Immunity.	global spectrum	systematic review
Wszolek KM; Chmaj-Wierzchowska K; Wilczak M	04-2021	Management of birth, postpartum care and breastfeeding - Polish recommendations and guidelines during SARS-CoV-2 pandemic.	Poland	review of recommendations
Yeo KT; Biswas A; Ho SKY; Kong JY; Bharadwaj S; Chinnadurai A; Yip WY; Ab Latiff NF; Quek BH; Yeo CL; Ng YPM; Ee KTT; Chua MC; Poon WB; Amin Z	04-2021	Guidance for the clinical management of infants born to mothers with suspected/confirmed COVID-19 in Singapore.	global spectrum	a query of research
Devarajan J; Chiang E; Cummings KC 3rd	05-2021	Pregnancy and delivery considerations during COVID-19.	global spectrum	a query of research
Ross-Davie M; Brodrick A; Randall W; Kerrigan A; McSherry M	06-2021	2. Labour and birth.	UK	a query of research
