# Supplementary material for: Protecting Breastfeeding during the COVID-19 Pandemic: A Scoping Review of Perinatal Care Recommendations in the Context of Maternal and Child Well-Being
Source: Int J Environ Res Public Health. 2022 Mar 11;19(6):3347. doi: 10.3390/ijerph19063347 (PMC8949921; doi:10.3390/ijerph19063347)
Supplement: Supplementary file 1 [file ijerph-19-03347-s001.zip › Supplementary Table S3.pdf]

Supplementary Table S3. Recommendations concerning the mode of delivery and companions on the labor for mothers with confirmed and/or suspected COVID-19.

Author and date of publication	Mode of delivery determined by obstetric indications	CS for severely ill patients	VD is preferred/ no contraindication to VD	Postpone/ delay delivery (if maternal and fetal conditions allow it)	COVID-19 is not an indication for preterm delivery	Use a mask throughout labor (mother)	Water births are contraindicated	Availability of support/companions during the labor	Limited/restricted visitation on labor and delivery	Screen visitors on the labor and delivery unit	Use of a mask by companions	Use of hands wash by companions	Support via video
Non-country specific													
Liang, 03-2020 [99]	+												
Poon, 04-2020 [36]	+	+											
Stephens, 04-2020 [58]	+							+	+	+			
Asadi, 04-2020	+	+											
Donders, 04-2020 [24]	+		+					+	+				
Narang, 05-2020 [37]	+	+						+	+	+			
Abdollahpour, 05- 2020 [25]		+	+					+	+				

TrapaniJúnior 06-2020 [26]		+	+	+				+	+	+	+	
Trevisanuto 06-2020 [62]	+											
Lavizzari 06-2020 [57]	+							+	+	+		
Ashokka 07-2020 [38]	+	+										
Goyal 07-2020 [39]	+	+						+	+	+	+	+
Api 07-2020 [52]	+				+							
Ryan 08-2020 [102]	+							+				
Mascarenhas 08-2020 [27]	+	+	+				+	+	+	+	+	
Czeresnia 09-2020 [28]			+	+			+					
Krupa 09-2020 [51]	+			+				+				
Góes 10-2020 [29]	+		+			+						
Barrero-Castillero 12-2021 [75]	+											
Kotlar 01-2021 [61]	+							+	+	+		

Pountoukidou 04-2021 [76]	+									
Devarajan 05-2021 [40]	+	+				+	+	+	+	
Australia										
Vogel 12-2020 [42]	+	+								
Brazil										
Stofel 08- 2020 [53]	+			+	+	+		+	+	
deOliveira 02-2021 [30]	+		+			+	+	+	+	+
Cardoso 02-2021 [31]	+	+	+			+	+	+	+	
China										
Chen 03-2020 [32]	+	+	+							
India										
Chawla 06-2020 [63]	+									
Sharma 08-2020 [33]	+	+	+	+						
Italy										
Franchi 03-2020 [43]		+								

Singh 11-2020 [34]	+	+	+								
Cavicchiolo 04-2021 [59]	+					+	+				
Giusti 04-2021 [44]	+	+				+	+	+			
Nigeria											
Okunade 07-2020 [45]	+	+				+	+				
Poland											
Kalinka 01-2021 [46]	+	+				+	+	+	+		
Wszolek 04-2021 [55]	+				+	+	+	+	+		
Russia											
Ignatko 05-2020 [47]	+	+		+		+	+	+		+	+
Saudi Arabia											
Faden 08-2020 [35]	+	+	+								
Spain											
López 06-2020 [56]	+				+	+		+	+		
Turkey											
Erdeve	+	+									

06-2020 [48]										
UK										
BMJ 05-2020 [54]	+			+	+	+	+			
Ross-Davie 03-2021 [49]	+	+	+		+	+		+		
USA										
Amatya 05-2020 [66]	+								+	
Boelig 05-2020 [60]	+				+	+	+			+
Boelig 10-2020 [50]	+	+			+	+	+			

[1] CS – cesarean section, [2] VD -vaginal delivery.
