# Supplementary material for: Protecting Breastfeeding during the COVID-19 Pandemic: A Scoping Review of Perinatal Care Recommendations in the Context of Maternal and Child Well-Being
Source: Int J Environ Res Public Health. 2022 Mar 11;19(6):3347. doi: 10.3390/ijerph19063347 (PMC8949921; doi:10.3390/ijerph19063347)
Supplement: Supplementary file 1 [file ijerph-19-03347-s001.zip › Supplementary Table S4.pdf]

Supplementary Table S4. Recommendations concerning visitor policies on maternity ward for mothers with confirmed and/or suspected COVID-19.

Author and date of publication	Screening visitors	Limit visits - no detailed recommendations	Limit the number of visitors to one	Visitors of a particular age only	Family members only	PPE[1] required	A healthy companion when the mother is severy ill	No visitors	Using video and teleconferences
					Global level				
Poon, 04-2020 [36]		+							
CalilVMLT, 04-2020 [64]			+						
Stephens, 04-2020 [58]	+		+					+	
Asadi, 04-2020 [41]		+							
Narang, 05-2020 [37]		+							
Abdollahpour, 05-2020 [25]		+							
TrapaniJúnior, 06-2020 [26]	+		+	+	+	+			
ShahbaziSighaldeh, 06-2020 [65]	+		+			+			
Trevisanuto, 06-2020 [62]			+		+				
Lavizzari, 06-2020 [57]		+						+	
Montes, 07-2020 [77]		+							
Api, 07-2020 [52]		+					+		
Czeresnia, 09-2020 [28]		+				+			
Krupa, 09-2020 [51]					+		+		
Góes, 10-2020 [29]	+			+	+	+			
Haiek, 01-2021 [72]									+
vanVeenendaal, 03-2021 [67]	+				+	+			+

Yeo, 04-2021 [73]		+							+
Devarajan, 05-2021 [40]								+	
Brazil									
deCarvalho, 05-2020 [70]				+				+	
deOliveira, 02-2021 [30]		+							
Cardoso, 02-2021 [31]				+					
China									
Chen, 03-2020 [32]		+							
Egypt									
Mostafa, 08-2020 [68]						+	+		
India									
Chawla, 06-2020 [63]	+		+		+				
Sachdeva, 08-2020 [69]					+	+			
Italy									
Davanzo, 03-2020 [101]								+	
Franchi, 03-2020 [43]								+	
Poland									
Wszolek, 04-2021 [55]								+	
Saudi Arabia									
Faden, 08-2020 [35]		+							
Spain									
López, 06-2020 [56]			+						
Russia									
Ignatko, 05-2020 [47]		+							
Turkey									
Erdeve, 06-2020 [48]		+							
UK									
Ross-Davie, 03-2021 [49]						+			
USA									
Boelig, 05-2020 [60]			+	+					
Amatya, 05-2020 [66]						+			+
Boelig, 10-2020 [50]	+		+						

[1] PPE - personal protective equipment.
