# Supplementary material for: Protecting Breastfeeding during the COVID-19 Pandemic: A Scoping Review of Perinatal Care Recommendations in the Context of Maternal and Child Well-Being
Source: Int J Environ Res Public Health. 2022 Mar 11;19(6):3347. doi: 10.3390/ijerph19063347 (PMC8949921; doi:10.3390/ijerph19063347)
Supplement: Supplementary file 1 [file ijerph-19-03347-s001.zip › Supplementary Table S5.pdf]

Supplementary Table S5. Recommendations concerning skin to skin contact for mothers with confirmed and/or suspected COVID-19.

Author and date of publication	SSC[1] is recommended - no mention about any additional precautions	SSC is possible with precautions directly mentioned (eg. surgical mask, hands disinfection)	Decision of applying SSC should be discussed with the woman (shared decision-making model)	SSC recommended in line with pre-pandemic recommendations (routine care)	SSC is not recommended or suspended	Suspension of SSC as an emerging issue and a problem	SSC possible on clinical and organizational conditions
Non-country specific level							
WHO, 04-2020 [78]		+					
CalilVMLT, 04- 2020 [64]					+		
Narang, 05-2020 [37]		+					+
Williams, 05-2020 [91]	+						
Tomori, 05-2020 [79]		+					
Pramana, 06-2020 [80]	+						
TrapaniJúnior, 06-2020 [26]					+		
Trevisanuto, 06-2020 [62]		+					+
Lavizzari, 06-2020 [57]	+		+				
Ashokka, 07-2020 [38]					+		
Choi, 08-2020 [81]	+						
Davanzo, 08-2020 [71]		+		+			
Mascarenhas, 08-2020 [27]					+		

NgYPM, 09-2020 [82]		+					+
Genoni, 09-2020 [83]		+					
Czeresnia, 09-2020 [28]		+					
Krupa, 09-2020 [51]		+					
Góes, 10-2020 [29]					+		
Dimopoulou, 11-2020 [84]	+		+				
Yeo, 11-2020 [95]			+				+
Barrero-Castillero, 12-2020 [75]		+					
VuHoang, 12-2020 [105]						+	
Haiek, 01-2021 [72]	+						
Kotlar, 01-2021 [61]		+					
Spatz, 02-2021 [85]	+			+		+	
Bartick, 03-2021 [86]	+					+	
vanVeenendaal, 03-2021 [67]	+						
Olonan-Jusi, 03-2021 [92]					+		
Yeo, 04-2021 [73]		+	+				+
Pountoukidou, 04-2021 [76]		+					+
Poon, 04-2020 [36]			+				
ShahbaziSighaldeh, 06-2020 [65]			+				
Australia							
Gribble, 11-2020 [89]		+		+		+	
Vogel, 12-2020 [42]		+		+			
Brazil							

deCarvalho, 05-2020 [70]					+		
Stofel, 08-2020 [53]		+					
deOliveira, 02-2021 [30]			+		+		+
Cardoso, 02-2021 [31]					+		
Gonçalves-Ferri, 03-2021 [94]				+	+	+	
Egypt							
Mostafa, 08-2020 [68]		+					
India							
Chawla, 06-2020 [63]					+		
Sharma, 08-2020 [33]		+					
Italy							
Giusti, 04-2021 [44]	+			+			
Cavicchiolo, 04-2021 [59]		+					
Japan							
Hosono, 04-2021 [93]					+		
Nigeria							
Ezenwa, 05-2020 [90]	+			+			
Poland							
Kalinka, 01-2021 [46]							+
Wszolek, 04-2021 [55]					+		
Spain							
López, 06-2020 [56]		+	+				+
Montes, 07-2020 [77]		+				+	
LalagunaMallada, 07-2020 [88]		+				+	

United Kingdom				
Ross-Davie, 03-2021 [49]	+	+	+	
USA				
Amatya, 05-2020 [66]				+
Harriel, 08-2020 [87]		+		
Boelig, 10-2020 [50]				+
Flannery, 04-2021 [74]		+		

[1] Skin to skin contact.
