# Supplementary material for: Protecting Breastfeeding during the COVID-19 Pandemic: A Scoping Review of Perinatal Care Recommendations in the Context of Maternal and Child Well-Being
Source: Int J Environ Res Public Health. 2022 Mar 11;19(6):3347. doi: 10.3390/ijerph19063347 (PMC8949921; doi:10.3390/ijerph19063347)
Supplement: Supplementary file 1 [file ijerph-19-03347-s001.zip › Supplementary Table S6.pdf]

Supplementary Table S6. Recommendations concerning rooming-in management for mothers with confirmed and/or suspected COVID-19.

Author and date of publication	Unconditional separation	Separation if COVID-19 diagnosed	Decision-based on the individual case	No separation, even if COVID-19 diagnosed	2 meters distance when COVID-19 diagnosed	2 meters distance independently from COVID-19 status
Non-country specific level						
Liang, 03-2020 [99]		+				
Poon, 04-2020 [36]		+			+	
CalilVMLT, 04-2020 [64]				+		+
Asadi, 04-2020 [41]		+				
Narang, 05-2020 [37]				+		
Williams, 05-2020 [91]				+		
Tomori, 05-2020 [79]				+		
Abdollahpour, 05-2020 [25]			+			
Pramana, 06-2020 [80]		+				
TrapaniJúnior, 06-2020 [26]				+		
Trevisanuto, 06-2020 [62]				+		
Lavizzari, 06-2020 [57]				+		
Api, 07-2020 [52]				+		
Choi, 08-2020 [81]				+		
Mascarenhas, 08-2020 [27]			+			
NgYPM, 09-2020 [82]				+		
Czeresnia, 09-2020 [28]		+				
Krupa, 09-2020 [51]				+		
Góes, 10-2020 [29]		+				+
Dimopoulou, 11-2020 [84]				+		
Yeo, 11-2020 [95]			+			+
Haiek, 01-2021 [72]				+		
Kotlar, 01-2021 [61]				+		
Spatz, 02-2021 [96]				+		
Olonan-Jusi, 03-2021 [92]			+			

Bartick, 03-2021 [86]				+		
vanVeenendaal, 03-2021 [67]				+		
Yeo, 04-2021 [73]			+		+	
Pountoukidou, 04-2021 [76]			+			
Australia						
Gribble, 11-2020 [89]				+		
Vogel, 12-2020 [42]				+		
Brazil						
deCarvalho, 05-2020 [70]				+		
deOliveira, 02-2021 [30]						+
Cardoso, 02-2021 [31]		+			+	
China						
Chen, 03-2020 [32]	+					
Egypt						
Mostafa, 08-2020 [68]			+	+		
India						
Chawla, 06-2020 [63]		+		+		
Sachdeva, 08-2020 [69]				+		
Sharma, 08-2020 [33]				+		
Italy						
Davanzo, 03-2020 [101]			+			
Franchi, 03-2020 [43]			+			
Moro, 11-2020 [100]		+				
Ronchi, 12-2020 [97]				+		
Giusti, 04-2021 [44]				+		
Nigeria						
Ezenwa, 05-2020 [90]			+			
Poland						
Kalinka, 01-2021 [46]				+		
Wszolek, 04-2021[55]				+		
Russia						
Ignatko, 05-2020 [47]	+					
Saudi Arabia						
Faden, 08-2020 [35]			+			

		Spain	
López, 06-2020 [56]			+
Montes, 07-2020 [77]			+
LalagunaMallada, 07-2020 [88]		+	
		Turkey	
Erdeve, 06-2020 [48]		+	
		UK	
Ross-Davie, 03-2021 [49]			+
		USA	
Boelig, 05-2020 [60]	+		
Amatya, 05-2020 [66]	+		
Harriel, 08-2020 [87]		+	
Perrine, 11-2020 [98]			+
